# The Telomere Binding Protein TRF2 Induces Chromatin Compaction

**DOI:** 10.1371/journal.pone.0019124

**Published:** 2011-04-19

**Authors:** Asmaa M. Baker, Qiang Fu, William Hayward, Samuel Victoria, Ilene M. Pedroso, Stuart M. Lindsay, Terace M. Fletcher

**Affiliations:** 1 Department of Biochemistry and Molecular Biology, University of Miami Miller School of Medicine, Miami, Florida, United States of America; 2 Department of Chemistry and Biochemistry, Arizona State University, Tempe, Arizona, United States of America; Tel Aviv University, Israel

## Abstract

Mammalian telomeres are specialized chromatin structures that require the telomere binding protein, TRF2, for maintaining chromosome stability. In addition to its ability to modulate DNA repair activities, TRF2 also has direct effects on DNA structure and topology. Given that mammalian telomeric chromatin includes nucleosomes, we investigated the effect of this protein on chromatin structure. TRF2 bound to reconstituted telomeric nucleosomal fibers through both its basic N-terminus and its C-terminal DNA binding domain. Analytical agarose gel electrophoresis (AAGE) studies showed that TRF2 promoted the folding of nucleosomal arrays into more compact structures by neutralizing negative surface charge. A construct containing the N-terminal and TRFH domains together altered the charge and radius of nucleosomal arrays similarly to full-length TRF2 suggesting that TRF2-driven changes in global chromatin structure were largely due to these regions. However, the most compact chromatin structures were induced by the isolated basic N-terminal region, as judged by both AAGE and atomic force microscopy. Although the N-terminal region condensed nucleosomal array fibers, the TRFH domain, known to alter DNA topology, was required for stimulation of a strand invasion-like reaction with nucleosomal arrays. Optimal strand invasion also required the C-terminal DNA binding domain. Furthermore, the reaction was not stimulated on linear histone-free DNA. Our data suggest that nucleosomal chromatin has the ability to facilitate this activity of TRF2 which is thought to be involved in stabilizing looped telomere structures.

## Introduction

The eukaryotic genome is packaged into complex nucleoprotein structures known as chromatin. The basic unit of chromatin structure is the core nucleosome, comprised of a histone octamer wrapped within 1.67 left-handed superhelical turns. Arrays of core nucleosomes are capable of folding into compact higher-order structures, a process facilitated by other chromatin architectural proteins such as linker histones. Any process that must occur on chromatin can potentially be modulated by its structure [1], [2], [3], [4], [5], [6], [7]. Conversely, factors that act upon chromatin may alter the structure of their chromatin substrates.

Telomeres, the ends of eukaryotic chromosomes, have a unique chromatin structure involving specific telomere binding proteins [8], [9]. In addition, mammalian telomeres have nucleosomes that are spaced more closely than bulk chromatin [9], [10], [11], [12]. Although nucleosomes appear to extend to the very end of the telomere [12], nucleosome saturation levels may depend on telomere length, as cell lines with shorter telomeres appear to have a lower histone density [13]. *In vitro* studies show that nucleosomes also slide more readily along telomeric DNA relative to nucleosome positioning sequences [14]. Together, these findings suggest that telomeric chromatin has unique properties.

Although some DNA damage signaling and repair proteins associate with telomeres during and/or immediately following their replication [15], [16], telomeres function to protect chromosome ends from being continually recognized as double-stranded DNA breaks throughout the rest of the cell cycle. Telomeres are maintained and protected by a DNA polymerase, telomerase, along with shelterin, a specialized complex(es) of six telomere binding proteins [8]. Knock-outs, knock-downs or dominant negative mutants of several shelterin proteins result in telomere dysfunction [17], [18], [19], [20], [21], [22], [23], [24], [25].

The two shelterin proteins that interact directly with double-stranded telomeric DNA are TTAGGG repeat factors 1 and 2 (TRF1 and TRF2), which bind via their homologous myb/SANT-like DNA binding domains (DBDs) [26], [27], [28], [29]. Both proteins negatively regulate telomere length [30]. Moreover, cells expressing a dominant-negative TRF2 have chromosome-end fusions with ensuing p53/ATM-mediated cellular senescence or apoptosis [18]. Conversely, TRF2 overexpression inhibits cellular senescence [31] and inactivates DNA damage checkpoint kinases, ATM [32] and Chk2 [33]. Interestingly, TRF2 overexpression in mouse keratinocytes also increases the frequency of skin tumors [34].

TRF2 interacts with and stabilizes different DNA architectures. For example, it stabilizes a telomeric DNA loop (t-loop) *in vitro* [35], [36], [37], a structure also isolated from cells of a variety of different species [35], [38], [39]. The ability to be both crosslinked by psoralen and bound by *E. coli* single-stranded DNA binding (ssb) protein at the t-loop junction suggests that the structure may be stabilized by a strand invasion reaction [35]. Thus, in addition to participating in the shelterin complex, TRF2 is thought to be the protein that sequesters chromosome ends from deleterious DNA processing by remodeling them into t-loops. At non-telomeric regions of the genome, TRF2 may also stimulate homologous recombination [40]. However, lacking unwinding or filament-forming activities, TRF2 probably does not stimulate a typical strand invasion on its own. Instead, TRF2 binds to DNA either through its myb/SANT domain [29] and/or its basic N-terminus [41] and alters DNA topology through its homodimerization domain (TRF homology or TRFH) [42]. This destabilizes the telomeric duplex on supercoiled DNA allowing for annealing of a telomeric oligonucleotide to form a displacement loop (D-loop) [43]. Furthermore, TRF2, via its highly-basic N-terminus, interacts with 4-way junction DNA [41], increasing its rate of formation and stabilizing it in a unique conformation, thereby inhibiting junction resolving activities [44]. The TRF2 N-terminus is also responsible for its interactions with telomeric RNA [45]. Furthermore, the N-terminus stabilizes G-quadruplex DNA secondary structures that inhibit t-loop reactions *in vitro* while inhibition can be overcome by the presence of the TRFH domain [46]. Interestingly, stimulation of this reaction occurs on reconstituted nucleosomal array fibers but not linear histone-free DNA [47]. All together, these studies suggest that TRF2 promotes t-loops by binding through more than one DNA binding motif while oligomerizing and altering DNA topology through its TRFH domain. This way TRF2 may directly stabilize the t-loop to sequester the 3′ end from damage signaling and prevent subsequent recombination steps that would result in loss of telomeric DNA from chromosomes. In fact, a dominant-negative TRF2 lacking the N-terminus triggers catastrophic loss of telomeres and the production of extra chromosomal telomeric DNA circles [19], [48].

Recent findings suggest that there is abundant TRF2 at telomeres [49] raising the possibility that TRF2 can influence chromatin structure. Although we have previously shown that TRF2 can alter chromatin structure through its Myb/SANT DNA binding domain [47], the effect of the TRFH domain and basic N-terminus on chromatin structure and stimulation of a t-loop reaction remained uncharacterized. In this study, we show that while TRF2 induced histone-free DNA self-association mainly through its basic N-terminus and TRFH domain, self-association is not efficiently induced on nucleosomal array fibers. Instead, the basic N-terminus neutralized negative charge on the surface of nucleosomal array fibers to stimulate their intrinsic ability to compact. Finally, the TRFH and Myb/SANT domains were required for optimal stimulation of a reaction associated with t-loop formation.

## Materials and Methods

### Materials

The DNA in these studies was derived from the 3.5 kb pRST5 plasmid [36] which contains ∼ 96, TTAGGG DNA repeats. The plasmid was digested with SfaNI to liberate a ∼2 kb fragment with the telomeric DNA in the center (Figure S1B). The plasmid was also digested with SfaNI/PvuII/BspHI (Figure S1B) to liberate a 1 kb telomeric fragment with smaller non-telomeric fragments to better observe TRF2-dependent mobility shifts of nucleosomal arrays. Fragments containing telomeric DNA were either purified from agarose gels or were left unpurified allowing for non-telomeric DNA to be used as an internal control for the AAGE analysis.

Recombinant, His6-tagged TRF2^DBD+L^ and TRF2^BH^ (Figure S1) were expressed in *E. coli* BL21(D3) cells (Invitrogen) and purified according to [46], [50]. The basic N-terminal region of TRF2 (TRF2^B^), containing amino acids 2–31 and an N-terminal biotin followed by a lysine (Biotin-KAGGGGSSDGSGRAAGRRASRSSGRARRGRH) was synthesized by Invitrogen. Recombinant, His6-tagged full-length TRF2 was baculovirus expressed in Sf9 cells and purified as previously described [51].

### Reconstitution of Nucleosomal Arrays

Histone octamers were purified from HeLa cells [52] or chicken erythrocytes [53]. Nucleosomal arrays were reconstituted by poly-L-glutamate transfer or stepwise salt dilution as described previously [47]. For AFM studies, nucleosomal arrays were reconstituted with chicken erythrocyte histone octamers by stepwise salt dialysis and dialyzed against 1 mM Na_2_EDTA (pH 8.0) overnight at 4°C as previously described [47]. Nucleosome saturation levels were analyzed by AAGE and AFM, and reconstitutes with high saturation levels (at least 1 nucleosome/200 bp) were used for experiments unless otherwise indicated.

### Micrococcal Nuclease Digestion

To verify proper reconstitution, an aliquot of reconstituted nucleosomal arrays (0.5 µg) was digested with 0.6 units/µl of micrococcal nuclease (Worthington) in 20 mM Tris-HCl and 2 mM CaCl_2_ (20 µl reaction volume). The reaction was stopped with a mixture of 5 mM Na_2_EDTA and 1% SDS and the DNA was separated on a 1.5% agarose gel.

### Formation of TRF2 Complexes with DNA or Nucleosomal Arrays for EMSA, Differential Centrifugation and AAGE

Indicated concentrations of TRF2 were incubated for 30 min at room temperature with 1.73 nM DNA or 2.71 nM reconstituted nucleosomal arrays (∼166 nM and 260 nM TTAGGG repeats respectively) in EMSA buffer (20 mM HEPES pH 7.8, 150 mM KCl, 0.5 mM MgCl_2_, 20% glycerol). Complexes were either detected by electrophoresis on 0.3% or 0.6% agarose gels in TAE (40 mM Tris-acetate, pH 8.0, 1 mM EDTA) running buffer and staining with SYBR Gold or analyzed by AAGE or differential centrifugation.

### Differential Centrifugation

TRF2 complexes with DNA or nucleosomal arrays (10 µl) were centrifuged in a microcentrifuge for 10 minutes at 16,000 rpm. The supernatant was removed and treated with 3 µg trypsin in 1% SDS at 37°C for 1 hour. Samples were then electrophoresed on 1% agarose gels. SYBR Gold-stained bands pertaining to telomeric DNA were quantified using ImageQuant software.

### Analytical Agarose Gel Electrophoresis (AAGE)

Multi-gels were poured using a specially designed apparatus (Aquabogue) and previously described method [47], [54], [55]. Agarose (Low EEO, Research Organics) concentrations within the multi-gels ranged from 0.25%–1.0%. Samples were prepared as described for binding experiments. Bromophenol blue/xylene cyanol loading dye was added to the samples which were loaded into the multi-gels and run for 3 hrs at 2 V/cm. Carboxylate-coated microspheres (35 nm radius, Duke Scientific) were added to the gels after 2 hrs running and samples were electrophoresed for the remaining hour. Gels were stained with SYBR Gold, imaged, and migrations were measured with ImageQuant software to obtain electrophoretic mobilities (µ) of DNA/nucleosomal arrays and microspheres.

The linear portion (0.25–1.0%) of a Ferguson plot (semi logarithmic plot of µ *vs.* agarose concentration) was extrapolated to 0% agarose to obtain the gel-free mobility (µ'_o_) for DNA, nucleosomal arrays and microspheres. The pore sizes of the gels (P_e_) and R_e_ for DNA or nucleosomal arrays (NA) and for each multi-gel experiment were calculated as described previously [47], [54], [55] using the R_e_ for microspheres (35 nm). The R_e_ values for DNA or NA were obtained by averaging R_e_ values from 0.25–0.6% gels in which no DNA reptation was observed.

### Atomic Force Microscopy (AFM)

Reconstituted nucleosomal arrays were crosslinked by dialyzing against 0.1% glutaraldehyde in 1 mM Na_2_EDTA (pH 8.0) for 6 hours at 4°C. All samples were first imaged by AFM to check histone octamer loading. Samples with high saturation levels were chosen for most experiments [47] except where indicated.

Histone-free DNA or nucleosomal arrays reconstituted with chicken erythrocyte histones were incubated for 30 min at room temperature with indicated concentrations of TRF2^B^ in EMSA buffer lacking both Mg^2+^ and KCl. The resulting complexes were crosslinked with 0.1% glutaradehyde for an additional 30 min, and diluted with EMSA buffer lacking Mg^2+^ to 0.3 ng/µl (in DNA) for imaging. A 10 µl aliquot of each sample was deposited on APTES-mica [56], pretreated with 2 µM glutaraldehyde, and incubated for 20 min, followed by rinsing with distilled water and drying with nitrogen. The imaging was carried out with a PicoPlus 2500+ (Molecular Imaging, 5500 AFM (N9410S) from Agilent) AFM equipped with a Si_3_N_4_ cantilever (AppNano SPM) and a spring constant range from 25–75 N/m. The resonance frequency was around 300 kHz; the scan rate was 1.71 Hz. Gwyddion and Chromatin Analysis 1.1.7 software was used for image analysis.

### Insertion of Single-Stranded Oligonucleotides into Nucleosomal Arrays or Histone-Free DNA (“Strand Invasion Reaction”)

The single-stranded DNA insertion assay was performed as described previously [43], [46], [47]. Nucleosomal arrays or histone-free DNA (200 ng), created using SfaNI digested PRST5, were incubated for 15 min at room temperature, in the presence of TRF2 or truncated mutants at specified concentrations, with 100 mM NaCl and reaction buffer containing 50 mM HEPES, 1 mM DTT and 2% glycerol. 5′-^32^P-labeled d(TTAGGG)_7_ oligonucleotide (T7) was added to a final concentration of 25 nM, and the reaction (10 µl total) was incubated for an additional 30 min. The reaction was stopped with 1% SDS (final) and 6 µg of proteinase K. After incubating for 1–2 hr, bromophenol blue loading dye was added and the samples were run on a 1.3% agarose gel in TBE (90 mM Tris-borate, pH 8.3, 2 mM EDTA). Radiolabeled oligonucleotide (free and inserted into plasmid) was detected by phosphorimaging and analyzed by ImageQuant software.

## Results

### TRF2 Binds to Chromatin and the TRF2 Basic N-Terminus and TRFH Domain Induce DNA Self-Association

TRF2 has been shown to alter DNA topology [43] and stimulate t-loop formation on histone-free DNA [35], [36]; activities that may be modulated by the presence of nucleosomes. To better understand how TRF2 performs this function in the context of chromatin, we analyzed the binding of TRF2 to fibers of nucleosomal arrays in comparison to histone-free DNA. Nucleosomal array fibers were reconstituted and characterized by micrococcal nuclease digestion, analytical agarose gel electrophoresis (AAGE) and atomic force microscopy (AFM) as previously described. As detailed in our earlier publication [47] nucleosomes were difficult to reconstitute on a 2 kb substrate with ∼600 bp of telomeric DNA in the middle possibly because telomeric nucleosomes are less stabile and slide readily along telomeric DNA [14]. Saturation required the poly-L-glutamate method [57] to more reproducibly obtain nucleosomal arrays that had at least 1 nucleosome/200 bp of DNA. Salt dialysis with higher histone:DNA ratios (1.3∶1, Figure S1C and D) than typically used in experiments with nucleosome positioning sequences [58] was also used. Nucleosomal arrays with a high level of saturation were used in most experiments except where indicated.

**Figure 1:**
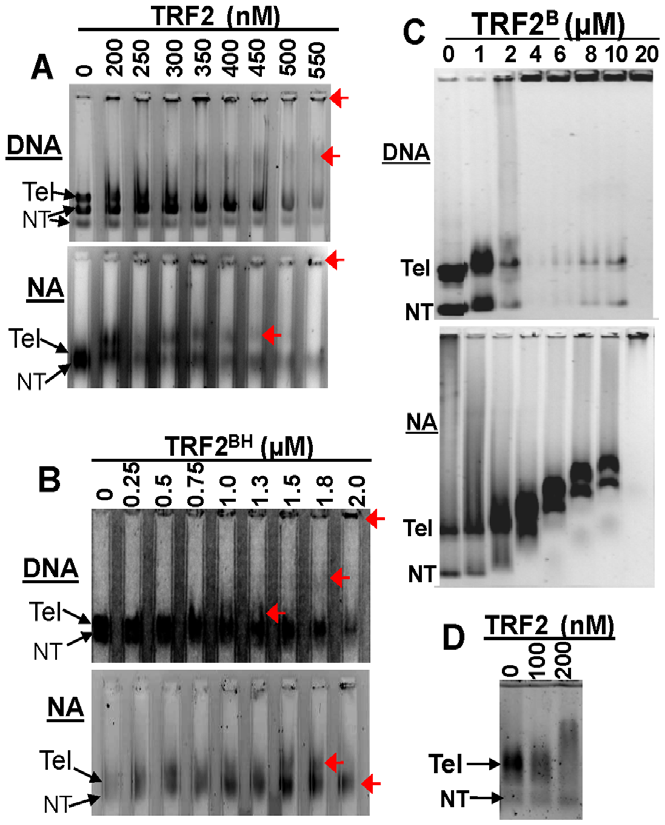
TRF2 binds to telomeric DNA (DNA) and nucleosomal array fibers (NA). TRF2 (**A**) or TRF2^BH^ (**B**) binding to substrates detected by electrophoresis on 0.3% agarose gels or 0.6% agarose gels to detect binding of TRF2^B^ (**C**). DNA and nucleosomal arrays pertain to pRST5 digested to obtain a 2 kb fragment containing the 580 bp telomeric DNA (Tel) with a 1 kb and smaller fragments being non-telomeric (NT). 0.6% agarose gel to detect binding of TRF2 to nucleosomal arrays derived from digestion of with SfaNI/PvuII/BspHI (**D**).The 0.3% agarose lanes in (A) and (B) were formed using a multi-gel apparatus as described in Materials and Methods. Red arrows point to mobility shifts produced by TRF2 or TRF2^BH^ complexes.

Next, TRF2 was incubated with either DNA or reconstituted nucleosomal array fibers and complexes were detected by agarose gel electrophoresis. It has been shown that TRF2 oligomerizes on DNA and brings sections of DNA together to form t-loops [35], [36], [37]. In agreement, we observed that TRF2 substantially reduced the mobility of telomeric DNA even in 0.3% agarose gels with the majority of the complexes not entering the gels (Figure 1A). Similar concentrations of TRF2 were required to alter the mobility of telomeric nucleosomal arrays (Figure 1A). However, binding of TRF2 to nucleosomal arrays produced a structure(s) with only slightly reduced mobility, while much higher TRF2 concentrations were required to shift the mobility of nucleosomal fibers into the wells (Figure 1A). When the pRST5 plasmid was digested with additional enzymes to obtain a 1 kb telomeric fragment with small non-telomeric fragments, binding of TRF2 produced a mobility shift with reconstituted telomeric nucleosomal fibers that was well separated from smaller non-telomeric fragments (Figure 1D). Therefore, the binding of full-length TRF2 was specific as it did not alter the mobility of non-telomeric substrates.

The TRF2-dependent reduction in mobility of both DNA and nucleosomal array fibers may involve oligomerization of TRF2 on the DNA, neutralization of DNA negative charges and/or DNA condensation. To aid in determining the mechanisms for TRF2-dependent changes in chromatin structure, we identified the regions of TRF2 (Figure S1A) involved in altering certain physical parameters of the DNA or nucleosomal fiber substrates. We previously showed that the myb/SANT DNA binding domain (TRF2^DBD^) did not appear to form large complexes with either DNA or chromatin [47]. This suggests that the TRF2-driven, DNA self-association does not reside in this region. The linker region was previously found to stimulate oligomers on small model telomeric ends [50]. We found that the linker region contributed slightly to self-association as judged by a construct containing both the DBD and linker region (TRF2^DBD+L^), but the effect was not specific to DNA (data not shown). We then focused on the arginine-rich, N-terminus because it has been shown to bind to and/or stabilize 4-way junction DNA [41], [44], G-quadruplexes [46] and telomeric RNA [45]. A peptide consisting of 30 amino acids of the N-terminus (TRF2^B^) induced the formation of large TRF2^B^-DNA complexes that did not enter a 0.6% agarose gel (Figure 1C). As expected, TRF2^B^ affected the mobility of both telomeric and non-telomeric DNA demonstrating non-specific interactions with DNA. Similar to the full-length protein, much more TRF2^B^ was required to form complexes with nucleosomal fibers that could not enter the gels (Figure 1C). Instead, the mobility gradually decreased with increasing TRF2^B^. Another important region of TRF2 is the TRFH, homodimerization, domain which is required for alteration of DNA topology and stimulation of displacement loops (D-loops) within supercoiled plasmid DNA [43]. A construct containing both the TRFH domain and the arginine-rich, N-terminus (TRF2^BH^) bound to DNA and chromatin (Figure 1B). TRF2^BH^ also affected the mobility of non-telomeric fragments, as expected due to binding through the basic N-terminus. Interestingly, this construct shifted the mobility of DNA and nucleosomal arrays in a similar manner to that of the full-length protein; DNA shifted into the wells while the mobility shifts of nucleosomal arrays were more subtle. Therefore, these results suggest that the differential mobility shifts of DNA and nucleosomal fibers caused by TRF2 reside in the N-terminus and TRFH domain.

**Figure 2:**
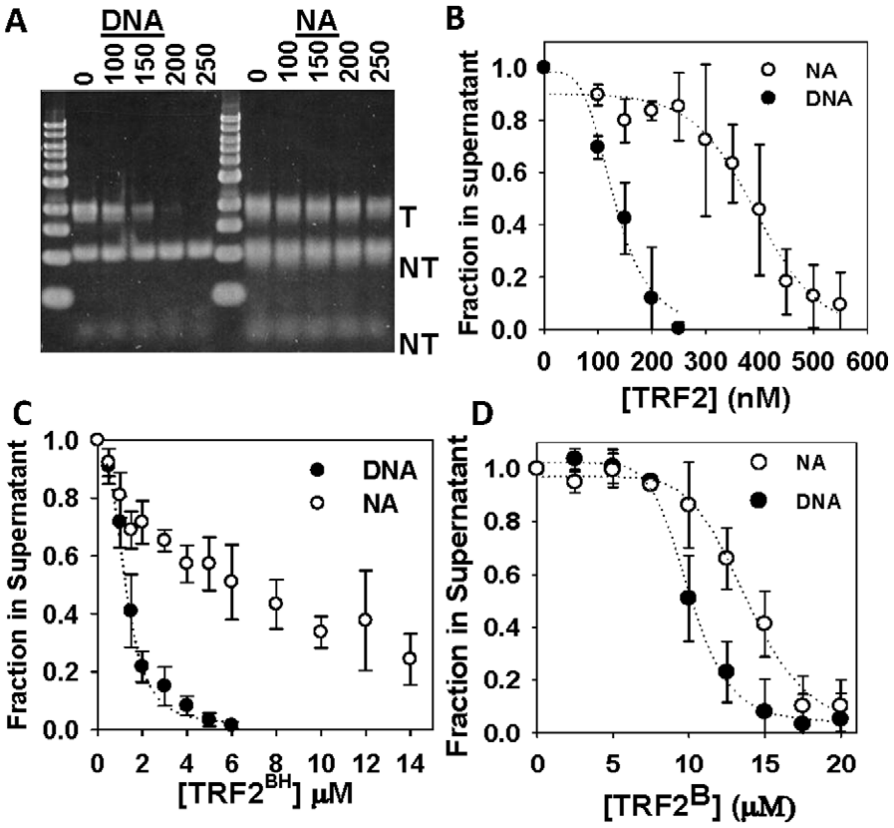
TRF2 stimulates self-association of DNA and nucleosomal arrays. Differential centrifugation assay as described in Materials and Methods. 1% agarose gel of samples with indicated amounts of TRF2 in nM where “T” refers to telomeric and “NT” refers to non-telomeric fragments (**A**). Quantification of experiments with TRF2 (**B**) TRF2^BH^ (**C**) and TRF2^B^ (**D**). Each data point represents the mean ± 1 SD from 3 separate experiments.

To quantify the amount of TRF2 required to induce self-association of DNA and nucleosomal array fibers, we employed a differential centrifugation assay (Figure 2). Salt-dependent chromatin self-association has been characterized by low speed sedimentation velocity experiments [59] and compared with differential centrifugation [5], [59], [60], [61], [62], [63], [64]. Using this assay, it was determined that nucleosomal arrays required ∼3 fold more TRF2 than histone-free DNA to form complexes large enough to be sedimented (Figure 2A). These results, together with gel electrophoresis demonstrate that although TRF2 binds readily to nucleosomal arrays, it does not induce self-association as it does with histone-free DNA.

Truncated forms of the protein were tested to determine where the DNA self-association activity resides. Like full-length TRF2, much more TRF2^BH^ was required to form large complexes with nucleosomal arrays compared to DNA (Figure 2B). Interestingly, DNA and nucleosomal arrays were sedimented with similar concentrations of TRF2^B^ (EC_50_ = 10–12 µM, Figure 2C). These results suggest that 2–10 µM TRF2^B^ was required to form well-shifted DNA species during electrophoresis but was not enough to form DNA complexes large enough to detect in the centrifugation assay. This also suggests that while DNA forms large complexes with 2–10 µM TRF2^B^, nucleosomal array fibers form different structures that still enter agarose gels. Only 20% of DNA and negligible amounts of nucleosomal arrays were sedimented with TRF2^DBD+L^ concentrations as high as 1 µM (data not shown). This is a 10-fold higher concentration than that needed to detect binding on agarose gels and suggests that the linker region does not contribute significantly to TRF2-driven, DNA self association. From mobility shift and differential centrifugation data, we conclude that the effects of TRF2 on DNA *vs.* nucleosomal array fibers described here reside mainly in the basic, N-terminus and TRFH domains.

### TRF2 Neutralizes Negative Charge and Induces Compaction of Nucleosomal Array Fibers

**Figure 3:**
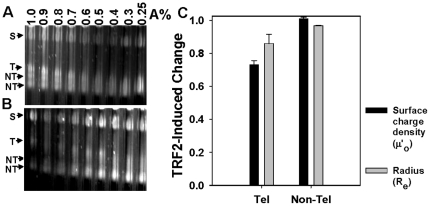
TRF2-dependent changes in surface charge density (µ'_o_) and effective radius (R_e_ from dilute gels) of nucleosomal fibers determined by analytical agarose gel electrophoresis (AAGE). Multi-gels of telomeric nucleosomal array fibers (NA) in the absence (**A**) or presence (**B**) of 200 nM TRF2 prepared and subjected to electrophoresis according to Materials and Methods. “S” refers to carboxylate-coated microsphere standards (35 nm radius). “T” refers to the telomeric fragments liberated by SfaNI/PvuII/BspHI digestion of pRST5 and “NT” refers to the non-telomeric DNA fragments. TRF2-induced change in surface charge density (µ'_o_) and effective radius (R_e_) of nucleosomal arrays derived from the telomeric (Tel) or non-telomeric (non-Tel) fragments (**C**). The µ'_o_ (black bars) or R_e_ (grey bars) of NA in the presence of 200 nM TRF2 was normalized to 0 nM TRF2. Bars represent the mean ±1 SD from 3 separate experiments. The data were derived from multi-gels of 0.25–1% agarose concentrations while the R_e_ bars represent the average from 0.25–0.6% agarose concentrations according to Materials and Methods.

The results in Figures 1 and 2 suggest that TRF2 forms complexes with DNA and nucleosomal arrays that have different structures. To further characterize the biophysical features of TRF2-induced structures, we used a method we term analytical agarose gel electrophoresis (AAGE). This method utilizes a multi-gel apparatus [47], [54], [55] to pour several agarose concentrations as dilute as 0.2% agarose to obtain accurate Ferguson plots, logarithm mobility (µ) as a function of agarose concentration. The y-intercept pertains to the gel-free mobility (µ'_o_) which is proportional to the surface electrical charge density [55]. It was difficult to obtain enough TRF2-DNA complexes that entered the agarose gels, limiting our analysis to nucleosomal array fibers. TRF2 (200 nM) reduced the negative surface charge of nucleosomal arrays by ∼ 30% (Figure 3), demonstrating that part of the reduction in electrophoretic mobility was due to neutralization of negative charge on the surface of the nucleosomal arrays.

Negative surface charge reduction could result from binding of a basic region of TRF2 along the nucleosomal arrays. Both the TRF2 N-terminus and DBD have positive charge available to neutralize negative charges on DNA. Charge neutralization could also be due to chromatin compaction which buries negative charge (with counterions) from the surface [65]. To observe compaction, the TRF2-dependent change in effective radius (R_e_) of the nucleosomal array fibers was determined by including microspheres of a known radius in each multi-gel experiment and utilizing a sieving equation [47], [54], [55]. Only dilute gels with pore sizes much larger than that of the nucleosomal fibers are used in these experiments to attain R_e_'s that reflect a radius similar to a Stoke's radius [55], [65]. This method has been used to detect the Mg^2+^-dependent folding of nucleosomal arrays [65]. TRF2 (200 nM) reduced the R_e_ of nucleosomal fibers (Figure 3) concomitant with the reduction in surface charge, raising the possibility that compaction may contribute to part of the reduction in negative charge. However, while TRF2 neutralized 30% of the negative surface charge, the reduction in R_e_ was less pronounced.

### Charge Neutralization by the TRF2 N-Terminus Induces Nucleosomal Fiber Compaction

**Figure 4:**
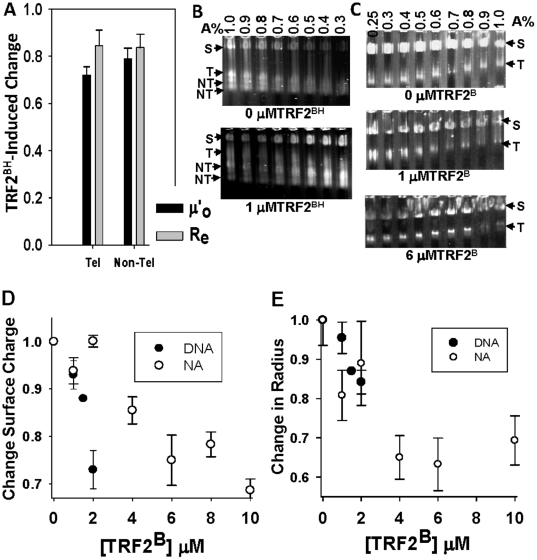
The role of the TRF2 basic N-terminus alone (TRF2^B^) or with the TRFH domain (TRF2^BH^) in TRF2-dependent negative charge reduction and compaction of nucleosomal arrays (NA). TRF2^BH^-induced change in surface charge density (µ'_o_) and effective radius (R_e_) of nucleosomal arrays derived from the telomeric (Tel) or largest non-telomeric (non-Tel) fragment (**A**). Bars represent the mean ±1 SD of 3 multi-gel experiments. The µ'_o_ (black bars) or R_e_ (grey bars) of NA in the presence of 1 µM TRF2^BH^ was normalized to 0 µM TRF2^BH^. Multi-gels of telomeric nucleosomal array fibers (NA) in the absence or presence of 1 µM TRF2^BH^ (**B**) prepared and subjected to electrophoresis according to Materials and Methods. “S” refers to carboxylate-coated microsphere standards (35 nm radius). “T” refers to the telomeric fragments liberated by SfaNI/PvuII/BspHI digestion of pRST5 and “NT” refers to the non-telomeric DNA fragments. Multi-gels of telomeric nucleosomal array fibers (NA) in the presence of indicated amounts of TRF2^B^ (**C**). TRF2^B^-induced changes in surface charge density (µ'_o_). (**D**) and effective radius (R_e_ from dilute gels) (**E**) of DNA and nucleosomal arrays (NA). The µ'_o_ or R_e_ for each TRF2^B^ concentration was normalized to 0 µM TRF2^B^. Each data point represents the mean ±1 SD of 3–4 multi-gel experiments.

To determine the mechanism of these structural changes, the role of key TRF2 regions in altering the structure of telomeric DNA and nucleosomal arrays was analyzed. We previously found that the TRF2^DBD^ reduced more negative charge on the surface of nucleosomal array fibers than DNA. Additional charge neutralization was not attributable to the linker region as judged by the inability of TRF2^DBD+L^ to reduce negative charge on either DNA or chromatin (data not shown). However, the N-terminal half of TRF2 (TRF2^BH^) containing both the basic, N-terminal region and the TRFH domain reduced the µ'_o_ and R_e_ of nucleosomal arrays in a similar manner to the full-length TRF2 (Figure 4A). TRF2^BH^ did differ from TRF2 in that it also reduced the µ'_o_ and R_e_ of non-telomeric DNA because the binding specificity for telomeric substrates resides in the DBD. Nevertheless, these results, together with those in Figures 1 and 2, suggest that the differential effects of TRF2 on the global structure and self-association of DNA and nucleosomal fibers reside within the basic, N-terminus and TRFH domain.

To further narrow down the region involved in compaction and to better observe compaction without the effect of TRFH-induced protein oligomerization, we performed AAGE experiments with TRF2^B^. DNA was analyzed as a comparison but analysis was limited to low concentrations of TRF2^B^ to prevent DNA self-association. TRF2^B^ neutralized ∼ 20% of the negative DNA surface charge at 2 µM (Figure 4D). Interestingly, TRF2^B^ also induced a slight decrease in the radius (∼10%) suggesting some level of DNA compaction (Figure 4E). TRF2^B^, having 8 arginines, is likely a multivalent cation and its effect on DNA may be similar to cooperative DNA collapse or condensation observed with binding of polyamines (*55*). The TRF2^B^ concentrations that promote DNA self-association had a different effect on nucleosomal array fibers. Neutralization of negative surface charge with 2–8 µM TRF2^B^ (Figure 4D) significantly reduced the R_e_ of nucleosomal array fibers (Figure 4E) suggesting that TRF^B^ promotes compaction. These fibers were considerably more compact than those in the presence of full-length TRF2 and TRF2^BH^. Moreover, the level of TRF2^B^-induced compaction was as expected if TRF2^B^ is a multivalent cation [66].

**Figure 5:**
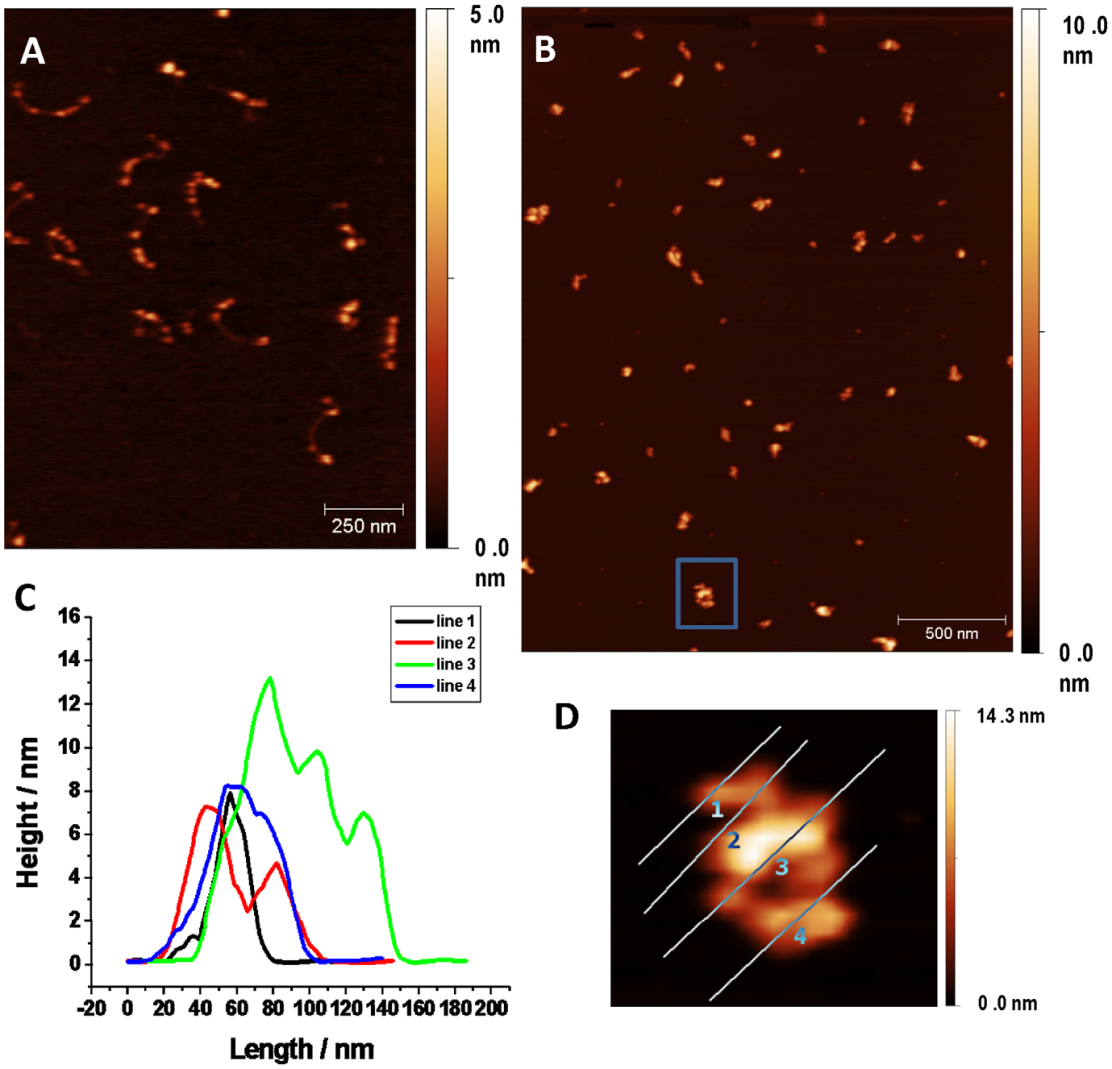
Atomic force microscopy of TRF2^B^-nucleosomal array complexes. Nucleosomal array fibers (reconstituted with 1∶1 histone:DNA mass ratio) in the absence of TRF2^B^ (**A**). Nucleosomal arrays with 4 µM TRF2^B^ (**B**). An example of height measurements (**C**) of regions indicated by lines drawn on the fiber (**D**) expanded from in the boxed region in (B). Samples were prepared and analyzed according to Materials and Methods.

Compaction was further validated by visualizing complexes by atomic force microscopy (Figure 5). In order to better view individual nucleosomes in the folded structures, we reconstituted fibers at both subsaturating and saturating histone:DNA ratios. In both reconstituted samples, individual nucleosomes were present along the extended fiber, with lower histone:DNA ratios providing slightly subsaturated nucleosomal arrays (Figure S1A, Figure S2A and Figure 5A). Lower concentrations of TRF2^B^ did not significantly alter the structure of the fibers (data not shown). However, as observed with AAGE analysis, fiber compaction was clearly evident at 4 µM TRF2^B^ (Figure 5B). Larger fiber diameters were indicated by an increase in fiber heights (Figure 5C and D and Figure S2). Furthermore, nucleosomes remained largely intact in the compacted structures (Figure 5B and D). Compaction of more saturated nucleosomal fibers with 4 µM TRF2^B^ was so pronounced that individual nucleosomes were more difficult to discern (Figure S3B and C). By 8 µM TRF2^B^, both saturated and subsaturated nucleosomal fibers had a similar level of compaction (Figure S4). TRF2^B^ (2 µM) also induced the formation of more condensed DNA structures, but the architecture was distinct from those formed by nucleosomal array fibers (data not shown). Taken together, our data shows that the TRF2 N-terminus promotes the intrinsic ability of nucleosomal arrays to fold into more compact structures by neutralizing negative surface charge.

Since TRF2 can bind to 4-way junction DNA [41], [44], it may promote compaction by interacting near the entry/exit points to form a chromatosome-like structure such as that observed with linker histones [67] and MeCP2 [68]. However, we observed no chromatosome-like structures that were resistant to micrococcal nuclease digestion (data not shown). Some smearing of the micrococcal nuclease ladder was observed, but only with high concentrations of TRF2 (>500 nM, data not shown).

### Insertion of Single-Stranded DNA into Telomeric Nucleosomal Fibers Is Stimulated by the TRF2 TRFH Domain

**Figure 6:**
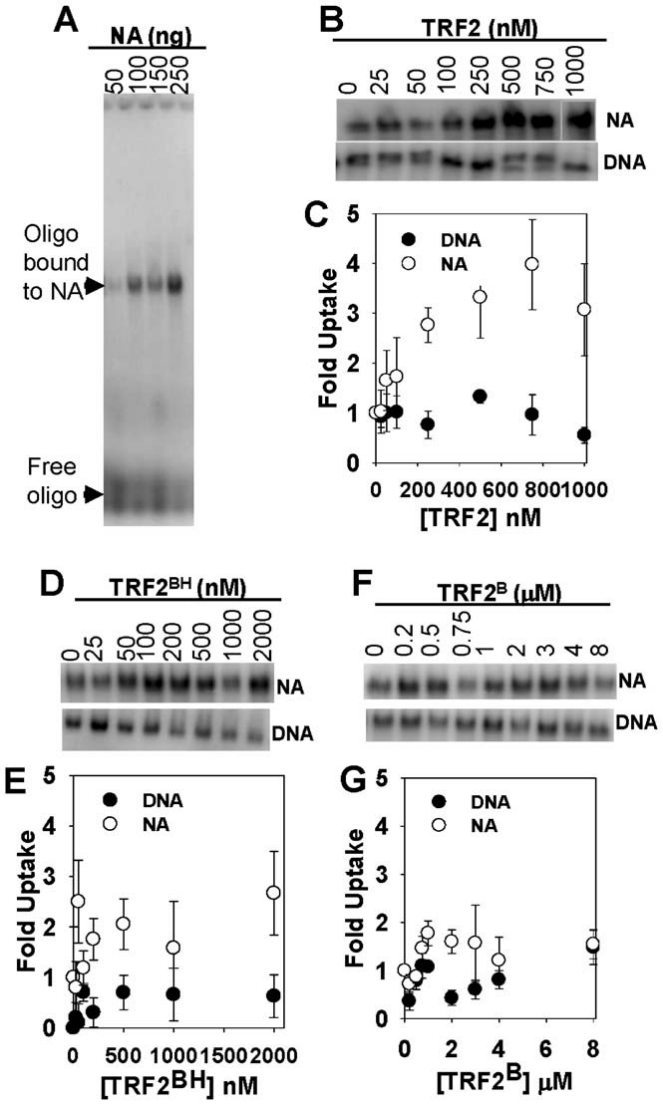
The effect of full-length TRF2, TRF2^BH^, and TRF2^B^ on the insertion of a 5′-[^32^P]-labeled, single-stranded oligonucleotide, (dTTAGGG)_7_ (T7), into nucleosomal arrays and DNA (20 ng/µl). Samples were incubated with indicated amounts TRF2 or its truncated mutants and processed according to Materials and Methods. Agarose gel showing insertion of T7 (Free oligo) into increasing amounts of nucleosomal arrays (Oligo bound to NA), as indicated (**A**). The section of agarose gels showing T7 inserted into nucleosomal arrays (NA) or linear DNA (DNA) with increasing TRF2 or truncation mutants as indicated (**B, D and F**). Quantification of corresponding gels above where uptake was normalized to 0 nM TRF2 or truncation mutants (**C, E and G**). Each data point represents the mean ±1 SD from 3–4 separate experiments.

TRF2 has been shown to stabilize t-loop structures *in vitro* [35], [36], [37], which have also been isolated from cells following psoralen crosslinking [35]. This structure has been proposed to protect chromosome ends by sequestering the 3′, G-strand overhang from spurious DNA metabolism and damage signaling. This is through formation of a displacement loop (D-loop) involving invasion of the G-strand overhang into the duplex region of the telomere. Telomeric D-loops are thought to form *in vitro* by insertion of a labeled single-stranded telomeric oligonucleotide (or 3′, single-stranded overhang) into a supercoiled, plasmid containing telomeric DNA in the presence of crude cellular extracts or recombinant TRF2 [16], [43]. This reaction requires telomeric sequence in both the single-stranded oligonucleotide and plasmid DNA [43]. TRF2 stimulates the reaction by generating positive superhelical density within the plasmid. We have reproduced the reaction by observing insertion of a telomeric single-strand oligonucleotide into nucleosomal fibers ([47] and Figure 6A). The reaction can be stimulated by TRF2 on nucleosomal fibers reconstituted onto linear DNA, while it is slightly inhibited on the corresponding histone-free DNA ([47] and Figure 6B and C).

The results in this study demonstrate that both TRF2^B^ and TRF2^BH^ alter the structure of telomeric nucleosomal arrays. To determine the effect of these structural changes in stimulating “strand invasion”, reactions were performed with TRF2^B^ and TRF2^BH^. It was previously found that the TRFH domain stimulated insertion of a telomeric oligonucleotide into a telomeric DNA plasmid by altering DNA topology [43], [69]. Consistent with this, we found that TRF2^BH^ slightly stimulated insertion of a telomeric oligonucleotide into nucleosomal array fibers (Figure 6D and E). However, less stimulation of the reaction was observed with TRF2^B^ (Figure 6F and G). Although we previously showed that the TRF2^DBD^ can also stimulate this reaction [47], none of these truncated forms were as effective as the full-length protein. Note that none of the TRF2 constructs could stimulate the reaction on linear histone-free DNA. These results, in addition to the previous study [47], suggest that that both the TRFH domain and DBD are involved in this strand-insertion reaction on nucleosomal array fibers while chromatin compaction per se has little effect.

## Discussion

The telomere binding protein, TRF2, is essential for maintaining the integrity of telomeres and stabilizing the genome. Considering the finding that there is enough TRF2 bound to nuclear chromatin to saturate telomeres [49], it is likely that TRF2 influences chromatin structure at telomeres if it can access nucleosomal chromatin. Furthermore, TRF2 has been known to alter DNA secondary structure and topology [43], [44], [46], [69]. Here, we show that TRF2 can access sites within nucleosomal fibers. However, TRF2 has distinct properties when bound to nucleosomal arrays compared to histone-free DNA; namely, it differs in the ability to neutralize negative charge on the substrate surface and induce either substrate self-association or compaction.

TRF2 forms complexes with DNA large enough to sediment in a microcentrifuge. These properties map partly to the basic N-terminus (TRF2^B^) with AAGE analysis showing significant negative surface charge neutralization and slight DNA compaction prior to DNA self-association. Electrophoretic mobility shift and differential centrifugation suggest that DNA condensation follows a path where DNA fragments self-associate stepwise to form structures large enough to be occluded from 0.6% gels but do not sediment until more TRF2^B^ is added (Figures 1 and 2). DNA self-association is also stimulated by the TRFH, homodimerization domain.

The processes that facilitate DNA self-association, instead promote the intrinsic folding of nucleosomal arrays into more compact structures similar to that observed with multivalent cations [66]. Our previous work [47] and this study demonstrate that both TRF2^B^ and higher concentrations of TRF2^DBD^ neutralize negative charge to induce chromatin folding. In this way, TRF2 has properties similar to chromatin architectural proteins [70]. However, unlike linker histones [67] or MeCP2 [68], TRF2 does not form a chromatosome structure with nucleosomes. Furthermore, other architectural proteins have little sequence specificity, while the TRF2^DBD^ targets the protein to telomeric sequence. Since TRF2 can interact with nucleosomal fibers in manners distinct from these other architectural proteins, it is possible that it can localize within telomeric chromatin that contains histone H1, along with core nucleosomes. However, this arrangement may be unique to longer telomeres since short telomeres of HeLa S3 cells appear to be deficient in H1 [13]. Moreover, TRF2 may recruit HP1 to telomeres mediated by its interactions with the telomeric RNA, TERRA [45].

Although the degree of compaction and charge neutralization can be attributed to the basic region and TRFH domain, these regions are not sufficient for optimal stimulation of the “strand invasion” reaction, as judged by insertion of a telomeric oligonucleotide into nucleosome arrays (Figure 6). This also requires TRF2 DBD, and we cannot rule out the possibility that the full-length protein is most efficient because the DBD targets it specifically to telomeric DNA. However, it is important to note that TRF2 only appears to stimulate the “strand invasion” reaction on either supercoiled plasmids [43], [47] or nucleosomal array fibers ([47] and Figure 6). Furthermore, TRF2 preferentially binds to positively-supercoiled DNA [43], [69] and it is thought that this induces duplex unwinding in topologically-constrained substrates such as plasmid DNA. We propose that something similar is occurring on nucleosomal fibers; TRF2 stimulates the t-loop reaction on nucleosomal array fibers by altering DNA topology through the TRFH domain which together with the DBD distorts and destabilizes the DNA duplex. This provides an opportunity for annealing of the oligonucleotide to form a D-loop.

While TRF2 promotes chromatin folding at lower concentrations, it is important to note that TRF2 has other properties that may affect nucleosomes if present in high concentrations. Smearing of the micrococcal nuclease ladder was observed with high concentrations of TRF2. Furthermore, we previously observed that the TRF2^DBD^ generates a slightly smeared micrococcal nuclease ladder while further addition of the protein creates a more compact structure that is inaccessible to the nuclease [47]. AAGE analysis also showed that even at low concentrations, TRF2^DBD^ could induce nucleosomal arrays to reptate through the pores of agarose gels [47], suggesting that it converts the usually rigid nucleosomal fiber rods into more conformationally flexible structures. This also suggests that TRF2 can distort the structure of nucleosomal arrays through its DBD in a manner distinct from its role in compaction. Furthermore, it has been shown that overexpression of TRF2 reduces the amount of histone H3 in telomeres of mouse keratinocytes, concomitant with an increase in nucleosome spacing [71]. TRF1, with its homologous DBD, can stimulate the intrinsic ability of nucleosomes to slide [14] when added at high concentrations [72] and induce DNase I hypersensitivity within the nucleosome at lower concentrations [73]. All together, the evidence suggests that both TRF2 and TRF1 affect telomeric chromatin in many ways without having to significantly displace histones.

The differential activities of TRF2 on DNA and nucleosomal substrates may also influence how TRF2 interacts with other proteins such as members of the shelterin complex [8]. Furthermore, TRF2 can alter activities of various DNA metabolic/repair enzymes and the ability of TRF2 to stimulate or inhibit certain activities depends on the nature of the substrate [44], [69], [74], [75], [76]. Although many of these activities likely occur at the replication fork where chromatin has been disrupted, TRF2 may facilitate replication by altering DNA topology [43], [69] and even influence chromatin assembly following the replication fork. These TRF2-dependent activities may be modulated by the surrounding chromatin environment.

## Supporting Information

Figure S1 **Domain structure of TRF2 and constructs discussed in this and previous** [**47**] **studies.** The N-terminal construct, TRF2^B^, was comprised of a peptide with the sequence, KAGGGGSSDGSGRAAGRRASRSSGRARRGRH, amino acids 1–31 of TRF2. TRF2^BH^ was derived from amino acids 1–246 of TRF2; TRF2^DBD+L^ was derived from amino acids 301–500 of TRF2; and TRF2^DBD^ was derived from amino acids 401–500 of TRF2 (**A**). DNA constructs used in this study were obtained by digesting the pRST5 plasmid with indicated enzymes. The telomeric DNA is indicated by the hatched rectangle (**B**). Multigels of DNA and nucleosomal array fibers derived from pRST5 digested with PvuII, SfaNI and BspHI (**C**). Atomic Force Microscopy of the 2 kb telomeric DNA fragment reconstituted with a 1.3∶1 histone:DNA mass ratio to obtain saturated nucleosomal array fibers (**D**).
(TIFF)

Figure S2 **Quantification of TRF2^B^-nucleosomal array fiber heights, obtained by atomic force microscopy.** Nucleosomal array fibers (reconstituted with 1∶1 histone:DNA mass ratio) in the absence of TRF2^B^ (**A**). Nucleosomal arrays with 4 µM TRF2^B^ (**B**). Histograms (C and D) representing heights obtained from (A) and (B) respectively. Samples were prepared and analyzed according to Materials and Methods.
(TIFF)

Figure S3 **Atomic force microscopy of TRF2^B^-nucleosomal array complexes using saturated nucleosomal arrays.** Nucleosomal array fibers (reconstituted with 1.3∶1 histone:DNA mass ratio) in the absence of TRF2^B^ (**A**). Nucleosomal arrays with 4 µM TRF2^B^ (**B**). Higher magnification of sample in (B) (**C**). Samples were prepared and analyzed according to Materials and Methods.
(TIFF)

Figure S4 **Atomic force microscopy of TRF2^B^-nucleosomal array complexes.** Nucleosomal array fibers, reconstituted with 1∶1 (**A**) or 1.3∶1 (**B**) histone:DNA mass ratio, in the presence of 8 µM TRF2^B^ (**B**). Samples were prepared and analyzed according to Materials and Methods.
(TIFF)
