# Supplementary figures and images for: The Telomere Binding Protein TRF2 Induces Chromatin Compaction
Source: PLoS One. 2011 Apr 19;6(4):e19124. doi: 10.1371/journal.pone.0019124 (PMC3079743; doi:10.1371/journal.pone.0019124)

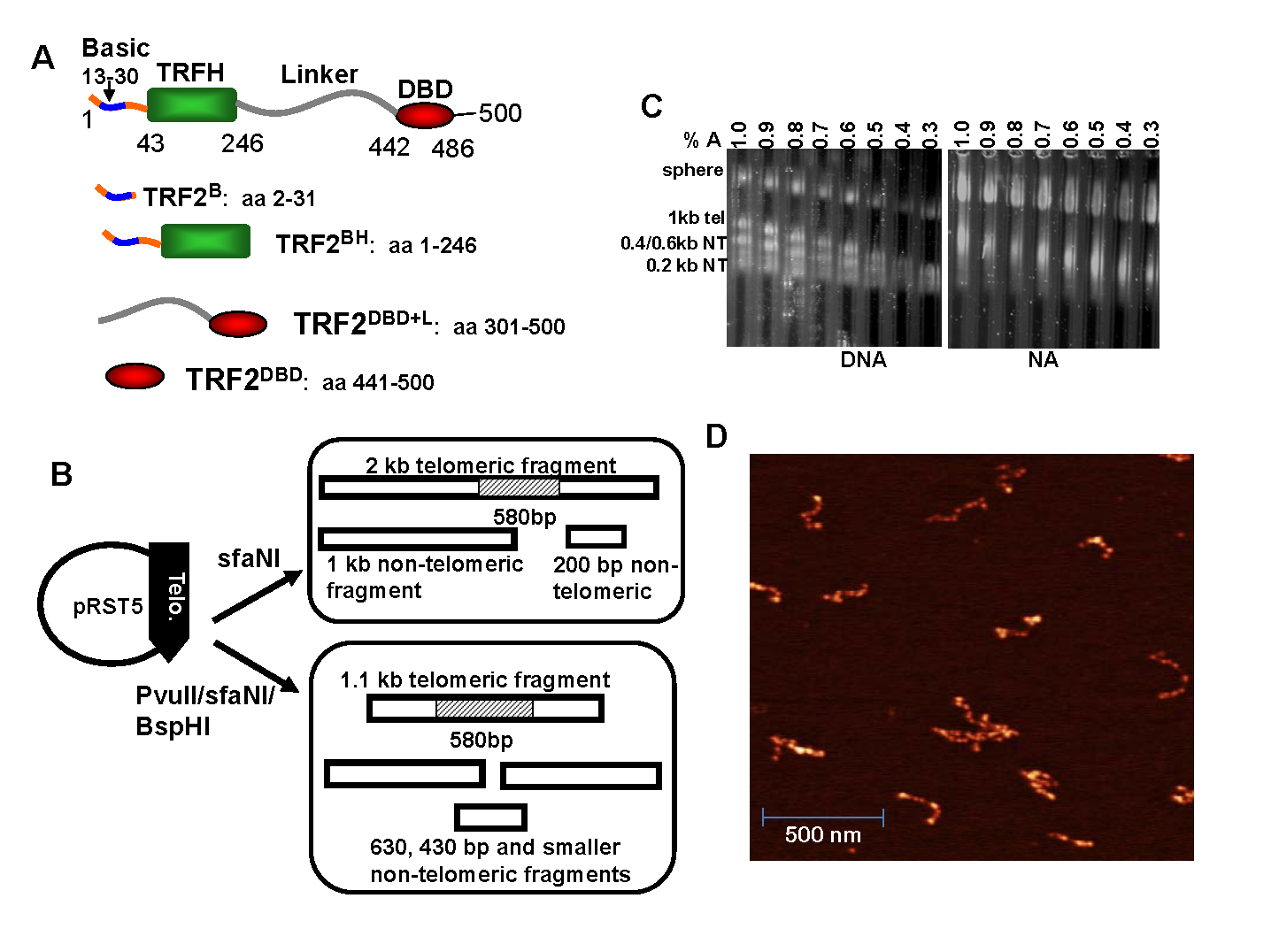

Supplement: Figure S1 — Domain structure of TRF2 and constructs discussed in this and previous [47] studies. The N-terminal construct, TRF2B, was comprised of a peptide with the sequence, KAGGGGSSDGSGRAAGRRASRSSGRARRGRH, amino acids 1–31 of TRF2. TRF2BH was derived from amino acids 1–246 of TRF2; TRF2DBD+L was derived from amino acids 301–500 of TRF2; and TRF2DBD was derived from amino acids 401–500 of TRF2 (A). DNA constructs used in this study were obtained by digesting the pRST5 plasmid with indicated enzymes. The telomeric DNA is indicated by the hatched rectangle (B). Multigels of DNA and nucleosomal array fibers derived from pRST5 digested with PvuII, SfaNI and BspHI (C). Atomic Force Microscopy of the 2 kb telomeric DNA fragment reconstituted with a 1.3∶1 histone:DNA mass ratio to obtain saturated nucleosomal array fibers (D). (TIFF) [file pone.0019124.s001.tif]

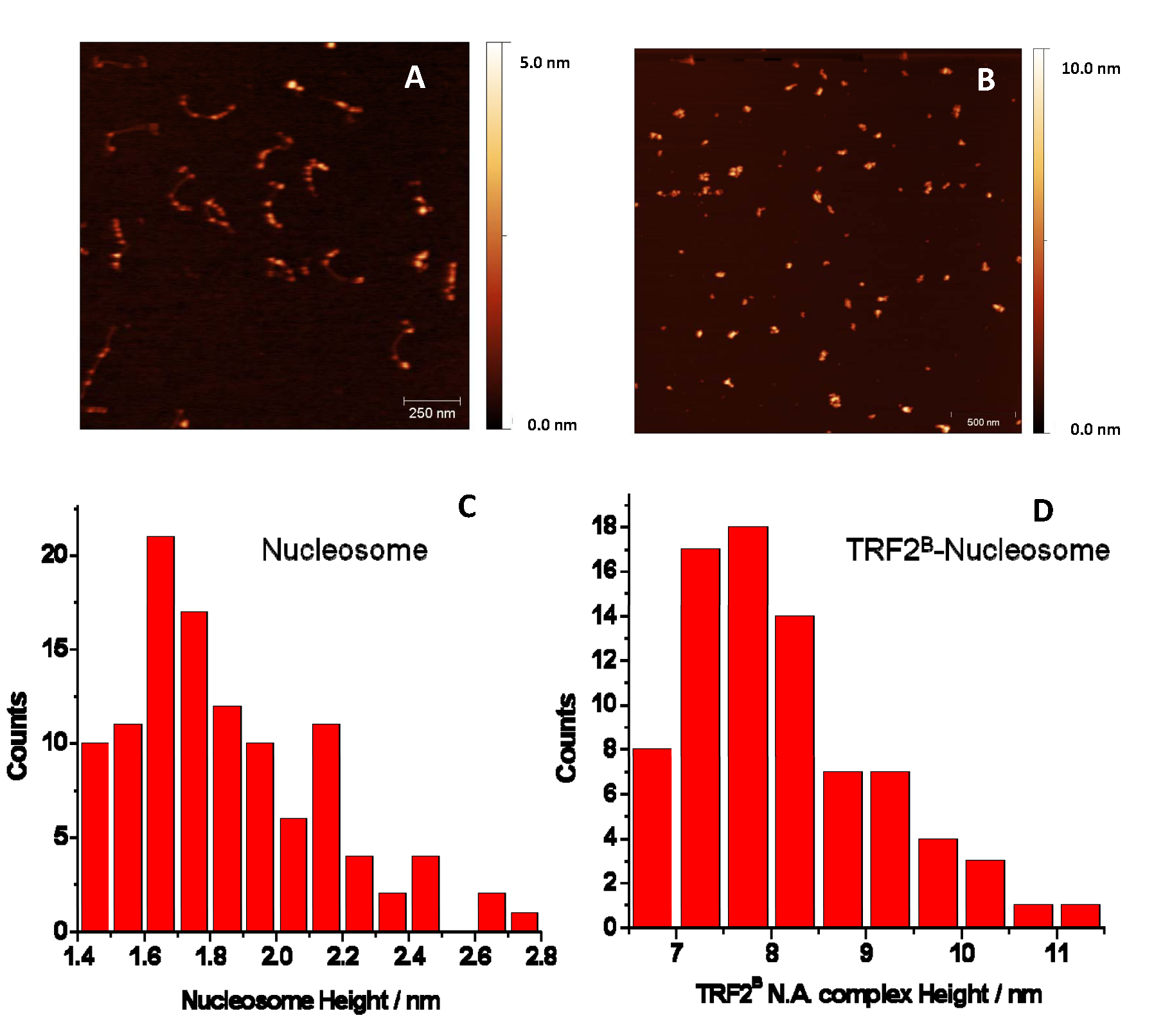

Supplement: Figure S2 — Quantification of TRF2B-nucleosomal array fiber heights, obtained by atomic force microscopy. Nucleosomal array fibers (reconstituted with 1∶1 histone:DNA mass ratio) in the absence of TRF2B (A). Nucleosomal arrays with 4 µM TRF2B (B). Histograms (C and D) representing heights obtained from (A) and (B) respectively. Samples were prepared and analyzed according to Materials and Methods. (TIFF) [file pone.0019124.s002.tif]

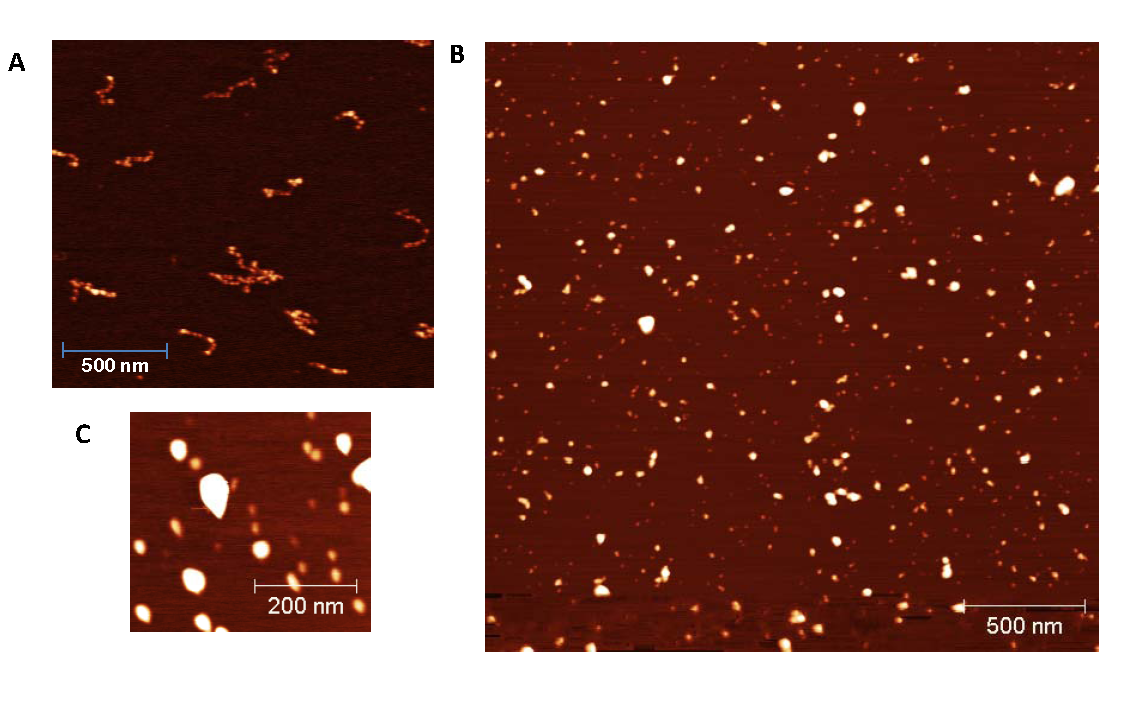

Supplement: Figure S3 — Atomic force microscopy of TRF2B-nucleosomal array complexes using saturated nucleosomal arrays. Nucleosomal array fibers (reconstituted with 1.3∶1 histone:DNA mass ratio) in the absence of TRF2B (A). Nucleosomal arrays with 4 µM TRF2B (B). Higher magnification of sample in (B) (C). Samples were prepared and analyzed according to Materials and Methods. (TIFF) [file pone.0019124.s003.tif]

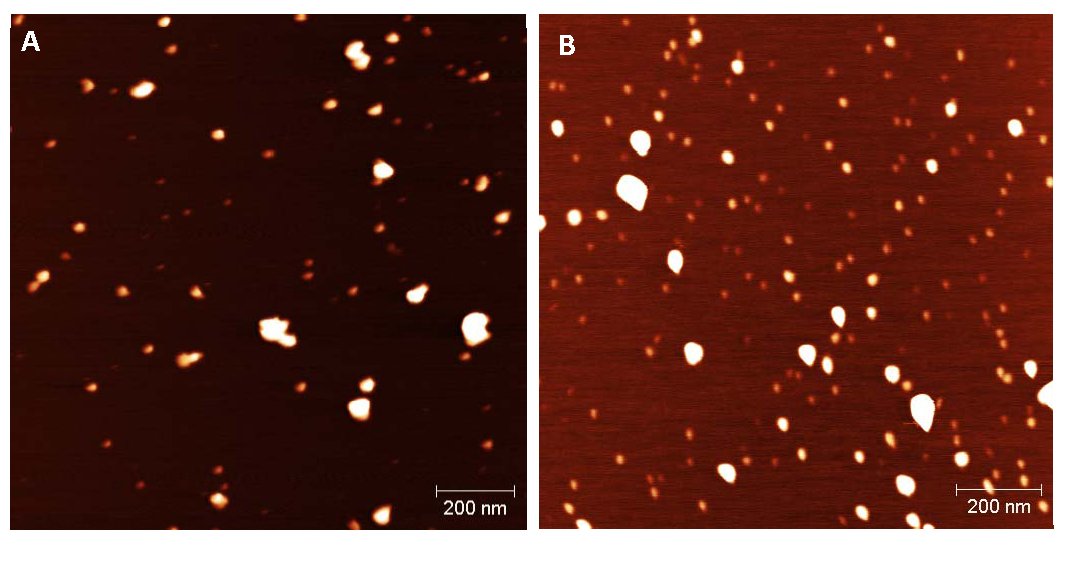

Supplement: Figure S4 — Atomic force microscopy of TRF2B-nucleosomal array complexes. Nucleosomal array fibers, reconstituted with 1∶1 (A) or 1.3∶1 (B) histone:DNA mass ratio, in the presence of 8 µM TRF2B (B). Samples were prepared and analyzed according to Materials and Methods. (TIFF) [file pone.0019124.s004.tif]
